# Effective *in vivo* therapeutic IgG antibody against VP3 of enterovirus 71 with receptor-competing activity

**DOI:** 10.1038/srep46402

**Published:** 2017-04-19

**Authors:** Qiang Jia, Qingyong Ng, Wenjie Chin, Tao Meng, Vincent Tak Kwong Chow, Cheng-I Wang, Jimmy Kwang, Fang He

**Affiliations:** 1 Animal Health Biotechnology, Temasek Life Sciences Laboratory, Republic of Singapore; 2 Singapore Immunology Network; Agency for Science; Technology and Research (A*STAR), Singapore; 3 Department of Microbiology and Immunology, Yong Loo Lin School of Medicine, National University of Singapore, Republic of Singapore; 4 Institute of Preventive Veterinary Medicine, and Zhejiang Provincial Key Laboratory of Preventive Veterinary Medicine, College of Animal Sciences, Zhejiang University, 866 Yuhangtang Road, Hangzhou, China

## Abstract

Passive immunization is an effective option for treatment against hand, foot and mouth disease caused by EV71, especially with cross-neutralizing IgG monoclonal antibodies. In this study, an EV71-specific IgG2a antibody designated 5H7 was identified and characterized. 5H7 efficiently neutralizes the major EV71 genogroups (A, B4, C2, C4). The conformational epitope of 5H7 was mapped to the highly conserved amino acid position 74 on VP3 capsid protein using escape mutants. Neutralization with 5H7 is mediated by the inhibition of viral attachment, as revealed by virus-binding and post-attachment assays. In a competitive pull-down assay with SCARB2, 5H7 blocks the receptor-binding site on EV71 for virus neutralization. Passive immunization of chimeric 5H7 protected 100% of two-week-old AG129 mice from lethal challenge with an EV71 B4 strain for both prophylactic and therapeutic treatments. In contrast, 10D3, a previously reported neutralizing antibody that takes effect after virus attachment, could only confer prophylactic protection. These results indicate that efficient interruption of viral attachment is critical for effective therapeutic activity with 5H7. This report documents a novel universal neutralizing IgG antibody for EV71 therapeutics and reveals the underlying mechanism.

Over the last decade, frequent epidemic outbreaks of hand, foot and mouth disease (HFMD) have been observed in the Asia-Pacific region. HFMD is mainly caused by human enterovirus 71 (EV71) and coxsackievirus A16. Severe disease and neurological complications are associated more often with EV71 infection, and can lead occasionally to fatal brain stem encephalitis in young children with rapidly developing symptoms^1^,^2^,^3^,^4^,^5^. In an outbreak of HFMD in 2008 in China, up to half a million cases were reported among children resulting in over 120 fatalities, which were primarily due to EV71 infection^6^. Also, an outbreak in 2012 in Cambodia led to the death of 54 children, most of them under 3 years of age. All samples obtained from fatal cases tested positive for EV71^7^(WHO: http://www.who.int/csr/don/2012_07_13/en/). Currently, putative inactivated vaccines are new in market early this year, and their efficacy in the community remains to be verified^8^. Prevention is mainly achieved by disrupting virus transmission with improved public hygiene in kindergartens, preschools and daycare centers aided by the temporary closures of affected places^9^. No specific treatment options exist so far^10^.

EV71 belongs to the human enterovirus A species (HEV-A) within the picornavirus family. The EV71 virion consists of a single-stranded positive-strand RNA of about 7.4 kb, surrounded by an icosahedral capsid composed of the four structural proteins VP1–4^11^,^12^. The viral RNA has a single open reading frame which is translated into a polyprotein upon cell entry, and is then cleaved auto-catalytically into the individual proteins. The polyprotein is divided into three regions, P1–P3. P1 encodes the structural proteins VP1–4. P2 and P3 span the seven non-structural proteins 2A–C and 3A–D. It is thought that the functions of these 11 proteins are identical to those described for poliovirus and other non-polio enteroviruses. While VP4 is found inside the virion with an extended conformation, the three major capsid proteins VP1, VP2 and VP3 form the outer surface of the virus^13^. To date, 11 subgenotypes (A, B10-B5 and C1-C5) have been identified based on the alignment of their VP1 sequences^14^. EV71-neutralizing antibodies are mainly elicited by VP1^15^,^16^ while only a few neutralizing epitopes have been identified in VP2^17^ and VP3^18^. Previously, the first conformational neutralizing epitope was identified in the knob region of EV71 VP3^19^, indicating the role of VP3 as a vaccine candidate or therapeutic target.

Human EV71-specific intravenous immunoglobulins are used for targeted treatment of severe cases^17^,^20^. However, besides the risk of transmitting human pathogens with the serum (necessitating screening and treatment), there are other disadvantages to using pooled human sera, e.g. the availability of donors and batch-to-batch variability^21^. Neutralizing monoclonal antibodies are attractive alternatives for passive immunization against EV71. Both effective therapeutic and prophylactic passive immunization against EV71 with neutralizing monoclonal antibodies in mice have been reported. Among these candidates, 10D3 is a broadly neutralizing antibody targeting VP3. However, the large-scale antibody production and humanization may be hindered by its IgM isotype, and its neutralizing mechanism was not elucidated. In this study, 5H7, an EV71 neutralizing IgG antibody was identified to target a new conformational epitope in VP3. Its efficacy as a therapeutic antibody was evaluated by EV71 lethal challenge in an AG129 mouse model^22^. The neutralization mechanisms of 5H7 and 10D3 were studied, and linked to their efficacy in EV71 treatment. A chimeric form of recombinant 5H7 was expressed, and its efficacy was further evaluated in AG129 mice upon EV71 infection.

## Materials and Methods

### Ethics statement

All animal experiments were carried out in accordance with the Guidelines for Animal Experiments of the National Institute of Infectious Diseases (NIID). Experimental protocols were reviewed and approved by Institutional Animal Care and Use Committee of the Temasek Life Sciences Laboratory, National University of Singapore, Singapore. (IACUC approval number TLL-14-015). Mice were housed in individually ventilated cages (Tecniplast Sealsafe), provided with water and standard chow, and monitored daily for health and clinical signs. More than 25% body weight loss was used as the criterion for early euthanasia. The animals were euthanized by CO_2_ inhalation for five minutes.

### Cells and viruses

African green monkey cell line (Vero ATCC number CCL-81) and human rhabdomyosarcoma cell line (RD ATCC number CCL-136) were obtained from American Type Culture Collection (ATCC) and grown in Dulbecco’s modified Eagle medium (DMEM, Gibco, USA) containing 10% fetal bovine serum (FBS, Biowest, France) at 37 °C with 5% CO_2_.

Wild-type (WT) EV71 strains and coxsackievirus A16 (CVA16) strain (U05876) were obtained from the Department of Microbiology and Immunology, Yong Loo Lin School of Medicine, National University of Singapore. The GenBank accession numbers of representative EV71 subgenogroups are listed in Table 1. Missing subgenogroups B1 (GenBank AF135901), B3 (GenBank AF376093) and C3 (GenBank AY125973) and confirmative mutants were constructed using the human RNA polymerase I reverse genetics (RG) system by inserting the relevant VP1 genes into the backbone of EV71 C4 strain Fuyang.Anhui.P.R.C/17.08/2 (GenBank EU703813). These viruses were propagated in RD cells grown in DMEM supplemented with 10% FBS at 37 °C with 5% CO_2_. Cell culture supernatants were harvested at 4 days post-infection (dpi). After three freeze-thaw cycles and filtration through a 0.2-μm cut-off filter (Sartorius, Germany), virus aliquots were stored at −80 °C. Virus activity was tested on RD cells in an end-point dilution assay to determine the 50% tissue culture infective dose (TCID_50_). EV71-B4 was inactivated with binary ethylenimine (BEI) for 48 h at 37 °C as described by Bahnemann^23^. For animal immunization, inactivated virus was concentrated 10-fold by ultracentrifugation at 100,000 g for 3 h and resuspended in PBS.

### Production and characterization of Mab

Three specific pathogen-free BALB/c mice were immunized subcutaneously on days 0, 14 and 28 with inactivated EV71-B4 strain in 0.1 ml PBS, emulsified with an equal volume of adjuvant (Seppic, France). An intraperitoneal booster of the same inactivated virus dose without adjuvant was administered 3 days before the mice were euthanized and their spleen cells harvested. Splenocytes were fused with SP2/0 myeloma cells as described, and cultured in DMEM with 20% FBS containing HAT or HT for 10 days. The hybridomas were screened by IFA in Vero cells infected with EV71-B4, subcloned by limiting dilution, and cultured. Selected positive Mabs were isotyped using a commercial isotyping kit (Amersham Bioscience, England), and were purified with Montage kit Prosep-G (Millipore) for IgG.

### Immunofluorescence assay (IFA)

RD or Vero cells were seeded into 96-well microtiter plates, and infected with a 10^−6^ dilution of EV71-B4. The cells were fixed at 2 dpi in 4% paraformaldehyde (pH 7.4) for 20 min, and permeabilized with 0.1% Triton-X/PBS for 30 min. The cells were incubated at 37 °C with hybridoma cell supernatants for 1 h. Anti-mouse FITC-coupled secondary antibody was then added for 1 h at 37 °C. Cells were washed three times with PBS between each step. Results were documented with an inverted fluorescence microscope (Olympus) with Nikon ACT-1 software.

### Virus neutralization assay

The neutralizing antibody titer was measured by an *in vitro* microneutralization assay using RD cells. 100 TCID_50_ of WT, escape mutant, or RG viruses were mixed with an equal volume of 2-fold serial dilutions of Mab 5H7 or control antibody. The mixtures were incubated for 1 h at room temperature before adding them in triplicates to the wells of microtiter plates containing 80% confluent RD cells. Presence of CPE was determined after 4 days by examination under light microscopy or IFA with anti-3CD antibody. The highest dilution of antibodies that inhibited virus growth was considered the neutralizing titer and expressed as 2^x^. Assays were conducted independently three times.

### Selection of escape mutants

The WT virus stocks EV71-A, EV71-B4, EV71-C2, and EV71-C4 were diluted to 100 TCID_50_ x neutralization titer, and incubated in an equal volume of neat Mab 5H7 (hybridoma supernatant) for 1 h at room temperature. The mixture was transferred to 80% confluent RD cells in DMEM with 10% FBS, and incubated for 4 days. If no CPE was observed, the supernatants were harvested, subjected to three freeze-thaw cycles, and filtered with a 0.2-μm cut-off before re-infecting a fresh batch of RD cells for 4 days. This was repeated until CPE was observed. One to three re-infection cycles were necessary for CPE, and hence EV71 escape mutants to develop. The escape mutants were designated E1-3/B4 (three individual experiments using EV71-B4 virus), E/A (EV71-A), E/C2 (EV71-C2), and E/C4 (EV71-C4). TCID_50_ was measured by end-point dilution and IFA. Microneutralization against Mab 5H7 was conducted to confirm abolishment of antibody binding and neutralization.

### Viral RNA isolation and cDNA sequencing

The QIAamp viral RNA isolation kit (Qiagen, Germany) was used to extract viral RNA from filtered RD cell culture supernatants containing WT and escape mutant virus. Typical yields were 80–100 ng/μL as measured by nanodrop (ThermoFisher Scientific, MA, USA). Reverse transcription was carried out on 500 ng RNA, using gene- and strain-specific primers together with Roche AMV reverse transcriptase (Roche Applied Science, Germany) according to the manufacturer’s protocol. PCR amplification of 2 overlapping portions of the P1 region was then conducted using the primer pairs (Table 2) and the High Fidelity PCR system (Roche Applied Science, Germany). The cycling parameters were as follows: denaturation at 94 °C for 2 min; followed by 10 cycles of denaturation at 94 °C for 30 sec, touchdown annealing from 54 °C to 45 °C in 1 °C decrements for 30 sec, extension at 72 °C for 2 min; followed by 30 cycles of denaturation at 94 °C for 30 sec, annealing at 45 °C for 30 sec, extension at 72 °C for 2 min + 5 sec per cycle increments and a final extension at 72 °C for 7 min. The PCR products were analyzed on 1% agarose gels, and purified by QIAquick gel extraction kit (Qiagen, Germany). Direct sequencing reactions were performed using gene- and strain-specific primers and BigDye terminator cycling at the DNA/Oligonucleotide Synthesis core of Temasek Life Sciences Laboratories, Singapore. Sequences were analyzed using the Lasergene programs (DNAstar, USA).

### 3D structure modeling

3D structure of EV71 C4 virus (MMDB ID: 97658)^8^ was downloaded from NCBI and viewed by the Cn3D 4.3 program. The views of either VP3 or other capsid proteins were generated to locate epitopes identified. The amino acid epitope recognized by 5H7 was highlighted in yellow at amino acid 74 on VP3, while the epitope of 10D3 was indicated by amino acids 59, 62 and 67. Other epitopes identified were highlighted in yellow in one protomer of the virus capsid. The mapped epitopes of EV71-specific Mabs 10D3 (VP3 59P, 62A and 67E), 7C7 (VP2 142-EDSHP-146), 51 (VP1 215-KQEKD-219) and 5H7 were presented.

### Construction of mutant viruses by reverse genetics

The genome of B4 wild type virus was amplified by RT-PCR and placed under the human RNA polymerase I promoter as described previously^24^. The infectious plasmids containing B4 cDNA (pJET-B4-wt) were sequenced and transfected to RD cells for virus generation. The mutations were introduced into the pJET-B4-wt plasmid by site-directed mutagenesis (Stratagene, CA, USA). Briefly, pJET-B4-P59L was mutated by primers B4-P59L-f (5′-GAGGTTAACAATGTACTCACCAATGCCACCAG-3′) and B4-P59L-r (5′-CTGGTGGCATTGGTGAGTACATTGTTAACCTC-3′), and pJET-B4-E67D by primers B4-E67D-f (5′-CACCAGTCTGATGGATAGGCTACGATTCCC-3′) and B4-E67D-r (5′-GGGAATCGTAGCCTATCCATCAGACTGGTG-3′). For double mutations in pJET-B4-PE59,67LD, pJET-B4-P59L was further mutated by primers B4-E67D-f and B4-E67D-r. The correctly mutated plasmids were transfected into RD cells for mutant generation, designated RG/B4-P59L, RG/B4-E67D, and RG/B4-PE59, 67LD, respectively.

### Western blotting

Cell lysates were separated by 12% sodium dodecyl sulfate-polyacrylamide gel electrophoresis. The proteins in the gel were transferred onto a nitrocellulose membrane, and blocked with 5% nonfat milk in PBST (1X PBS and 0.1% Tween 20) for 1 h at room temperature. The membrane was incubated with primary antibodies, rinsed with PBST, and incubated with horseradish peroxidase-conjugated rabbit anti-mouse or anti-human immunoglobulin G (IgG) (Dako, Denmark) for 1 h at room temperature. Following washing with PBST, the membrane was developed by incubation with ECL reagents (Amersham Biosciences).

### Virus binding assays

Vero cells were seeded into a T25 flask, and incubated overnight at 37 °C with 5% CO_2_. 2 ml of EV71 B4 virus (10^8^ TCID_50_/ml) mixed with either 2 ml of antibodies or medium were added to each T25 flask with Vero cells, and incubated at 37 °C for 1 hour. The mixture was removed after incubation. The flasks were washed 3 times with chilled DMEM. After one freeze-thaw round, the cells were scraped and subjected to sonication in 500 μl PBS. The lysate was clarified by centrifugation at 3900 g for 30 minutes. The supernatant was collected and tested by Western blot with Mab 57, an antibody against EV71 VP2.

### Post-attachment assays

Vero cells were seeded into a 96-well plate, and incubated overnight at 37 °C with 5% CO_2_. 100 TCID_50_ per 50 μl of EV71 B4 virus was added to each well, and incubated at 4 °C for 1 hour. After the virus was removed, the plate was washed 3 times with chilled DMEM. Antibodies were added to each well individually, and incubated at 37 °C for 1 hour. After the antibodies were removed, the plate was washed 3 times gently with DMEM, and incubated at 37 °C with 5% CO_2_ for 72 h. Infection of cells was tested by IFA with 4B12, an antibody against 3CD.

### Pull-down assays

5 μg of recombinant human SCARB2 (Catalog 1966-LM-50, R&D) was mixed with 15 μl of anti-human Fc beads (Catalog A3316-5 ml, Sigma Aldrich) and 30 μl of 1% milk. The mixture was incubated at 4 °C overnight, and subsequently washed 3 times with PBS. 20 μl of purified B4 virus (10^10^ TCID_50_/ml) was incubated with the respective amount of 5H7 Mab at 37 °C for 1 h. The Mab-B4 mixture was later added to the SCARB2-Fc beads or complexes, and incubated for another 2 h at 4 °C. B4-SCARB2-Fc beads were washed 5 times with PBS, and resuspended in PBS. The prepared samples were tested by SDS-PAGE and Western blot.

### Antigen-capture-ELISA and competitive ELISA

For AC-ELISA, U-bottomed 96-well ELISA plates were coated with purified Mab 57 (500 ng/well), and incubated overnight at 4 °C in coating buffer (0.1 mol/L carbonate/bicarbonate, pH 9.6). Coated plates were washed with PBS (pH 7.5) containing 0.05% Tween 20 (PBST), and nonspecific sites were blocked with 100 ul blocking buffer (PBST containing 5% skim milk) for 40 min at 37 °C. Virus samples were added to each well and incubated for 1 h at 37 °C.

For competitive ELISA, plates were coated with recombinant human SCARB2 (1 μg/well, Catalog 1966-LM-50, R&D) and blocked. 50 μl of purified B4 virus (10^10^ TCID_50_/ml) was incubated with antibody samples for 0.5 h at 37 °C, added to the blocked plate and incubated for 1 h at 37 °C.

In both ELISAs, purified anti-B4 guinea pig polyclonal antibody in PBST with 1% skim milk (400 ng/well) was added and incubated for 1 h at 37 °C. The wells were rinsed four times with PBST and incubated with horseradish peroxidase-conjugated secondary anti-guinea pig antibody (1:1000) Dako, Denmark) for 1 h at 37 °C. The wells were rinsed and incubated with 3, 39, 5, 59-tetramethyl benzidine (TMB, Sigma, USA). The reaction was stopped by 0.1 M sulfuric acid and the optical density (OD) was determined at 450 nm using a multiwell plate reader.

### Chimeric antibody construction

The heavy and light chain variable sequences were cloned from the total RNA of the hybridoma as described (http://www.ncbi.nlm.nih.gov/pubmed/10648866). The VL and VH sequences were subsequently fused to human kappa and IgG1 constant regions, respectively, to complete the expression constructs in two separate plasmids under the control of the CMV promoter. The resulting mouse-human chimeric antibody was expressed by transient transfection using Invitrogen 293fectin following the manufacturer’s instructions, and purified by Protein G resin (Minipore).

### Animal studies

The animal experiments were conducted using two-week old AG129 mice obtained from B&K Universal (UK). They were housed and bred under specific pathogen-free conditions in individual ventilated cages. Each group included 10 mice (with 5 mice in each group in the therapeutic study with chimeric antibody). Prophylactic groups of mice were injected intraperitoneally with purified antibodies (0.1 ml in PBS) at a concentration of 10 or 0 μg per g of body weight at 24 h before the challenge. Therapeutic groups were injected intraperitoneally with purified antibodies (0.1 ml in PBS) at a concentration of 10 or 0 μg per g of body weight at 24 h after the challenge. Mice in each group were subjected to a lethal challenge with 10^7^ plaque-forming units (PFU) of EV71 strain HFM41 (5865/SIN/00009) via the intraperitoneal route (0.4 ml in PBS). Survival rates and health scores of the mice were monitored daily till 20 dpi. The grade of clinical disease was scored as follows: 0, healthy; 1, lethargy and inactivity; 2, wasting; 3, limb weakness; 4, hindlimb paralysis or blindness; and 5, moribund and death.

### Histopathological analysis

Brain samples for histological examination were harvested from each group of mice. The tissues were soaked in 10% buffered formalin (pH 7.4), embedded in paraffin and sectioned at 5 μm thickness. The sections were de-paraffinized using Hist-choice (Amersco) and rehydrated in sequentially graduated ethanol baths. Immunohistochemistry (IHC) technique was used to detect the presence of viral antigen using 4B12, an antibody targeting the 3D polymerase of EV71 virus. Slides were de-paraffinized with Histo-clear II (National Diagnostics, Georgia, USA), and rehydrated in sequentially graduated ethanol baths. Slides were subjected to treatment according to the manufacturer’s instructions (DAKO animal research kit). The pathological evaluation was performed by light microscopy (Olympus, UK), and the images were captured by digital imaging system (Nikon, USA).

## Results

### 5H7 is a universal neutralizing antibody against EV71

**Figure 1:**
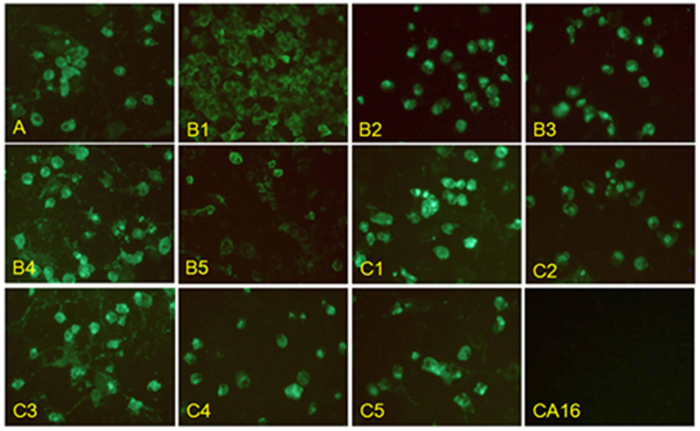
Mab 5H7 recognizes all 11 EV71 subgenogroups and does not cross-react with coxsackievirus A16. IFA of Vero cells infected with heterologous EV71 virus strains and coxsackievirus A16 (GenBank U05876). Cytopathic effects were visible at 2 days post-infection of cells fixed and labeled with supernatant of Mab 5H7-secreting hybridoma cells. FITC-conjugated anti-mouse Mab was used to detect viral signals. While all 11 EV71 subgenogroups were recognized by Mab 5H7, no signal was observed for CVA16. Magnification: 100×.

5H7 was generated from mice immunized with EV71-B4 strain. The 5H7 immunoglobulin isotype was determined as IgG2a. 5H7 was selected based on its broad reactivity to all 11 EV71 subgenogroups by IFA with Vero cells, without cross-reactivity to CVA16 (Fig. 1). Furthermore, 5H7 was able to neutralize all EV71 subgenogroups tested, without reactivity to CVA16 (Table 1). 5H7 failed to react to either EV71 whole virus or recombinant EV71 P1 polyprotein fragments by Western blot (data not shown), indicating a conformational epitope for the antibody. The results indicate that 5H7 is an EV71-specific broad neutralizing antibody.

### 5H7 targets a conserved conformational epitope containing 74S within VP3 capsid protein

**Figure 2:**
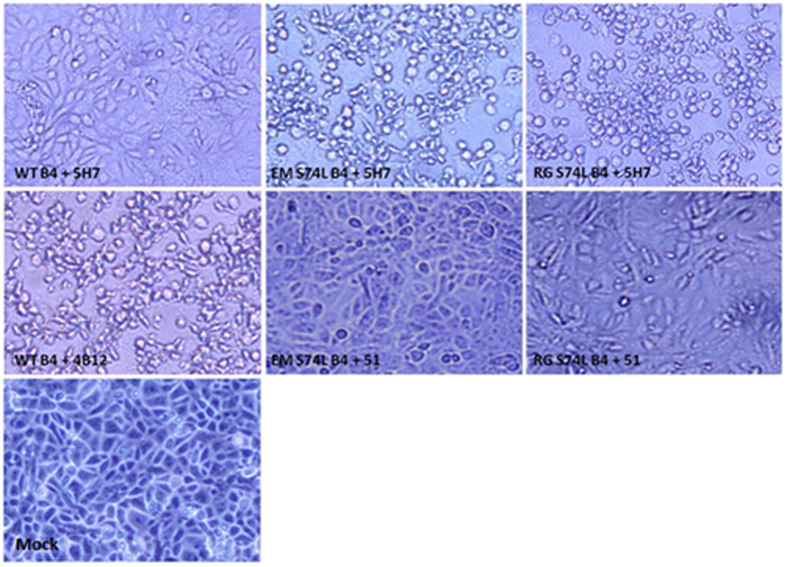
S74L mutation of VP3 confers evasion from neutralization with 5H7. Virus infection was indicated by CPE observed in inoculated Vero cells. No CPE was observed when neutralization occurred. RG: virus generated by reverse genetics; WT: wild-type virus; EM: escape mutant. 4B12: a non-neutralizing antibody against EV71 3CD; 51: a neutralizing antibody against VP1. Magnification: 100×.

The conformational epitope of 5H7 was mapped by escape mutant selection. Wild-type EV71 viruses from different subgenogroups (A, B4, C2, C4) were incubated with an excess of 5H7 in RD cells. The escape mutants were tested for reactivity with Mab 5H7 by IFA with parental viruses as controls. After incubation with 5H7, there were clear signals for parental viruses, but no signals for the corresponding escape mutants. To further confirm the evasion from 5H7 neutralization, a microneutralization assay against 100 TCID_50_ of each escaped virus was conducted. No neutralization activity by Mab 5H7 was detected against the identified escape mutants (Table 1 and Fig. 2).

**Figure 3:**
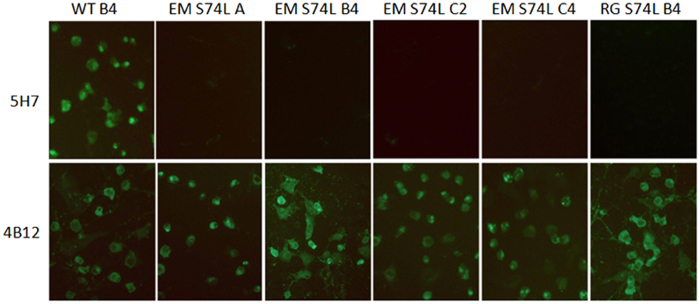
S74L escape mutation of VP3 protein abolishes 5H7 binding. Escape mutants of 5H7 were created by incubating EV71 B4 strain with an excess of Mab. Vero cells were then fixed and labeled with 5H7, followed by FITC-coupled secondary antibody. Cells infected with wild-type viruses served as positive controls. Mab 5H7 staining was abolished in the escape mutant carrying the mutation S74L (second row). 4B12, an antibody against EV71 3CD, was used as positive control for infection. RG: virus generated by reverse genetics; WT: wild-type virus; EM: escape mutant. Magnification: 100×.

**Figure 4:**
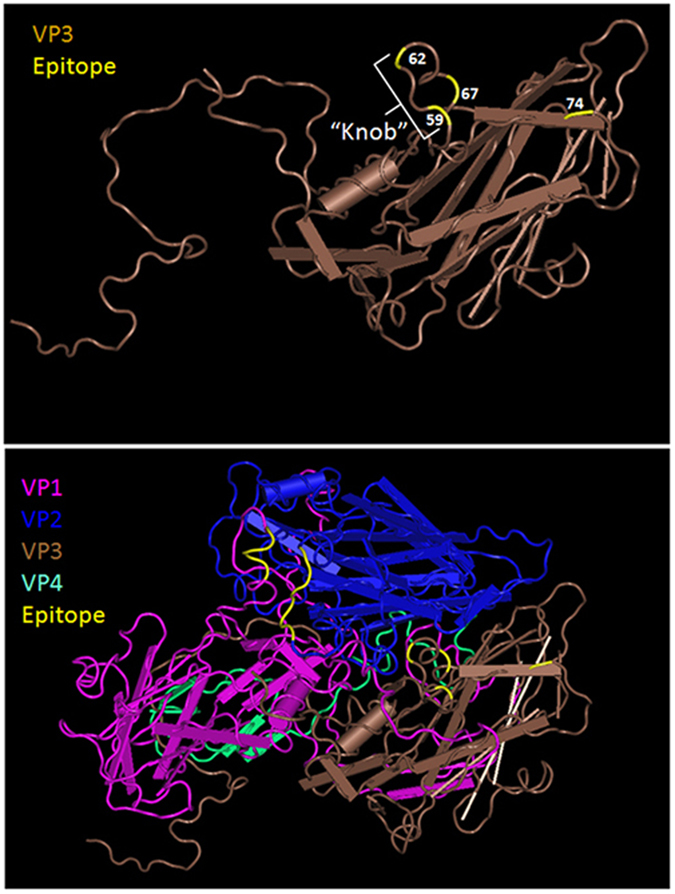
Stereographic images of 5H7 and 10D3 epitopes on VP3. The 3D crystal structure of EV71 C4 virus (MMDB ID: 97658) was downloaded from NCBI and viewed in the Cn3D program. (**A**) VP3 protein. EV71 VP3 protein is shown as a side-on view with the outside of the virion located at the top of the image and the inside on the bottom. 5H7 epitope is indicated as amino acid 74. 10D3 epitope is indicated in the “knob” structure as amino acids 55 to 69 of VP3. The escape mutation sites are indicated in yellow. (**B**) An EV71 protomer of the virus capsid is shown, containing one of each of the viral capsid proteins VP1 (pink), VP2 (blue), VP3 (brown), and VP4 (green). The mapped epitopes of EV71-specific Mabs 10D3 (VP3 59P, 62A and 67E), 7C7 (VP2 142-EDSHP-146) and 51 (VP1 215-KQEKD-219) as well as the escape mutation site of 5H7 at VP3 74S are indicated in yellow (arrows).

To elucidate the amino acid mutations associated with neutralization evasion, the P1 structural gene region of each escape mutant was sequenced and compared to its parental strain. One mutation was identified in the structural gene VP3 of each sub-genotype. All of the mutants derived from the parental strains harbored a serine-to-leucine substitution at amino acid position 74 of VP3 (Table 2). No other mutation was found. To confirm the neutralization evasion with the single mutation, a EV71-B4 virus consisting of the S74L mutation was constructed by utilizing a human RNA polymerase I driven RG system^24^. As shown by either IFA or neutralization (Table 1, Figs 2 and 3), Mab 5H7 failed to react with or neutralize the mutated RG virus, whereas the parental B4 was clearly detected and efficiently neutralized by 5H7. Hence, these results indicate that the single mutation S74L in VP3 is sufficient for the abolishment of 5H7 activity by virus detection and neutralization. Interestingly, the S74L mutation site is adjacent to the “knob” region on VP3 (Fig. 4), which represents the neutralizing epitope of 10D3 previously identified as a broad neutralizing IgM antibody against EV71.

### 5H7 disrupts virus attachment for neutralization

**Figure 5:**
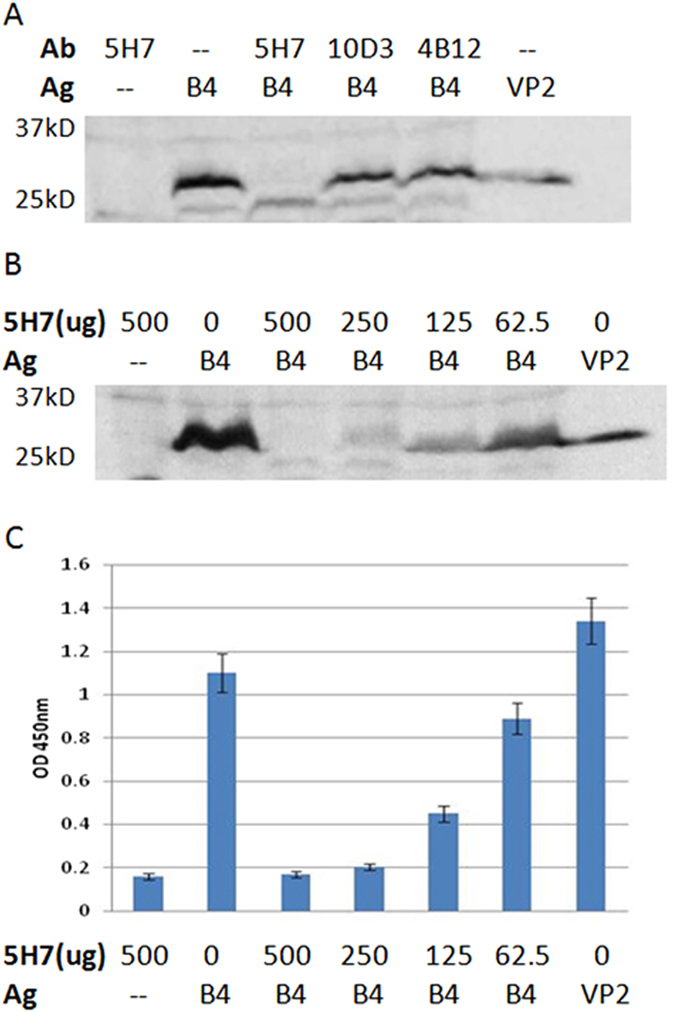
5H7 inhibits EV71 virus attachment. Virus-binding assays were performed in Vero cells with B4 EV71 virus. Cells were washed with PBS and lysed 1 hour after virus inoculation. Cell lysate was analyzed by Western blot with Mab 57 against VP2. (**A**) Binding assays detected in Western blot were performed with different EV71 antibodies individually (500 μg of each antibody). Cropped blots were presented. No viral protein was detected in the sample incubated with 5H7, indicating its inhibitory effect against EV71 attachment. 4B12: a non-neutralizing antibody against EV71 3CD; VP2: purified VP2 protein as the control for Western blot. (**B**) Binding assays detected in Western blot were performed with different amounts of 5H7. 250 μg of 5H7 was sufficient to neutralize 2 × 10^8^ TCID_50_ of B4 viruses. (**C**) Binding assays detected in antigen-capture ELISA were performed with different amounts of 5H7. No viral protein was detected in the samples incubated with at least 250 μg of 5H7.

Virus binding assays were performed to determine the effect of the antibodies on virus attachment. Results of Western blot (Fig. 5A) indicated that preincubation of 5H7 with EV71 B4 virus successfully prevented virus attachment to cells, whereas 10D3 failed to do so. A gradient of varying amounts of 5H7 was further tested to verify the inhibitory effect on virus attachment (Fig. 5B and C) by both Western blot and AC-ELISA. The results indicated that 250 μg of 5H7 was sufficient to neutralize 2 × 10^7^ TCID_50_ of virus for cell attachment.

**Figure 6:**
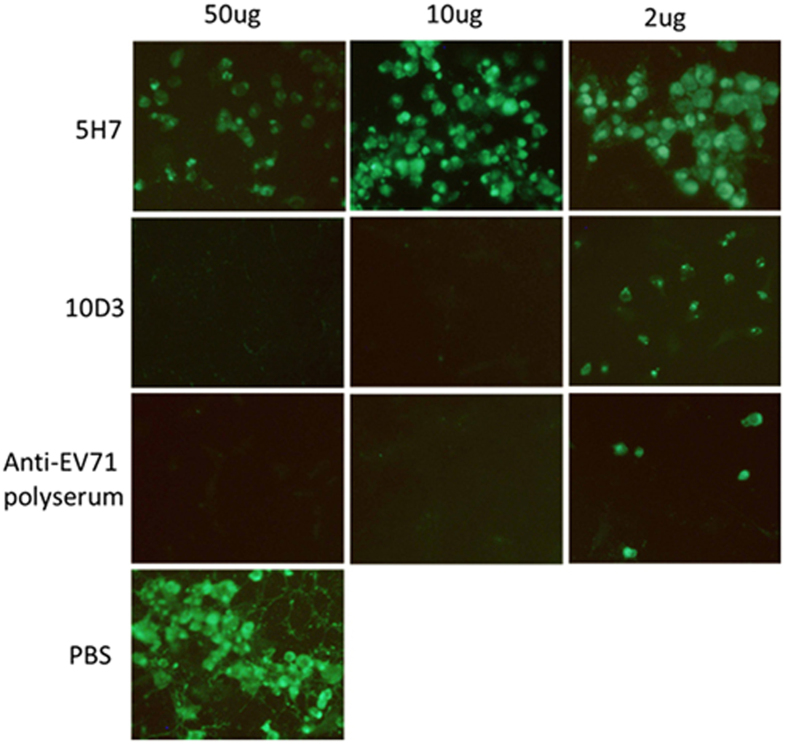
10D3 neutralizes EV71 post-attachment. Post-attachment neutralization assays were performed in Vero cells with varying amounts of 5H7, 10D3 and anti-EV71 polyserum (positive control). Virus infection was detected by IFA with 4B12, an antibody against 3CD. PBS indicated a control for B4 infection. Magnification: 100×.

Further, post-attachment neutralization was tested with 5H7 and 10D3 against B4 virus. Antibodies were inoculated to cells following virus entry. As shown in Fig. 6, 10D3 abolished virus expression indicating successful neutralization, whereas 5H7 failed to inhibit viral expression. This result confirmed that perturbing virus attachment is the sole neutralizing mechanism of 5H7, in contrast to 10D3 whose neutralizing effect occurs in steps after virus attachment.

**Figure 7:**
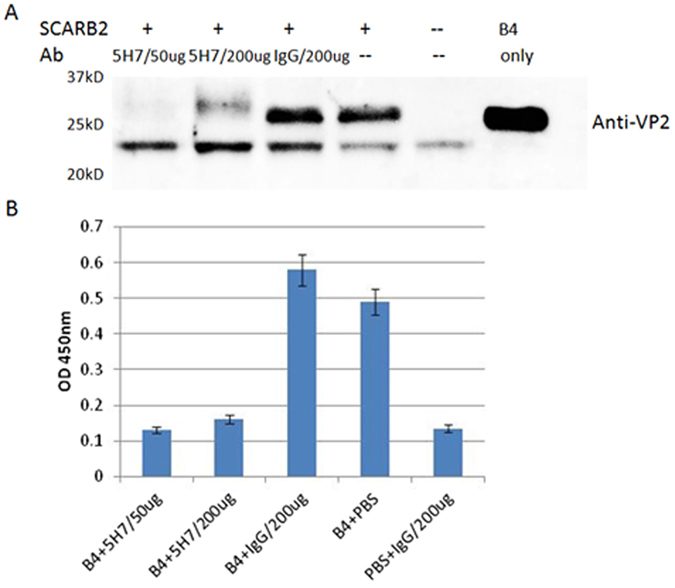
5H7 prevents EV71 from binding to SCARB2 receptor. (**A**): A pull-down assay was performed with SCARB2, B4 virus, and different antibodies. Virus binding to beads was detected by Western blot with Mab 57 against VP2. Cropped blots were presented. No virus band was detected in the samples incubated with 5H7. IgG: a non-EV71 IgG antibody served as a negative control. B4 only: a control for Western blot. (**B**) Competitive ELISA was also performed with SCARB2, B4 virus, and different antibodies. B4 virus was incubated with different antibodies, and added to wells coated with SCARB2. Virus binding to the coated wells was detected by guinea pig polyserum against B4. No virus was detected in the samples incubated with 5H7. IgG: a non-EV71 IgG antibody acted as a negative control.

To clarify the mechanism, a competitive pull-down assay (Fig. 7A) was performed with 5H7, B4 virus and SCARB2, the identified receptor for EV71^25^. In the presence of 5H7, beads conjugated with SCARB2 failed to bind to B4 virus. In contrast, SCARB2 beads were able to pull-down B4 viruses with a non-EV71 IgG antibody or without antibody. Similarly, by competitive ELISA (Fig. 7B), B4 virus failed to bind to SCARB2 in the presence of 5H7, whereas B4 virus incubated with other control antibodies was captured and detected by the same ELISA. These findings revealed that 5H7 prevents EV71 from binding to SCARB2 receptor, thereby leading to failure of virus attachment, and successful virus neutralization.

### 5H7 therapeutic strategy protects mice from lethal challenge with EV71

**Figure 8:**
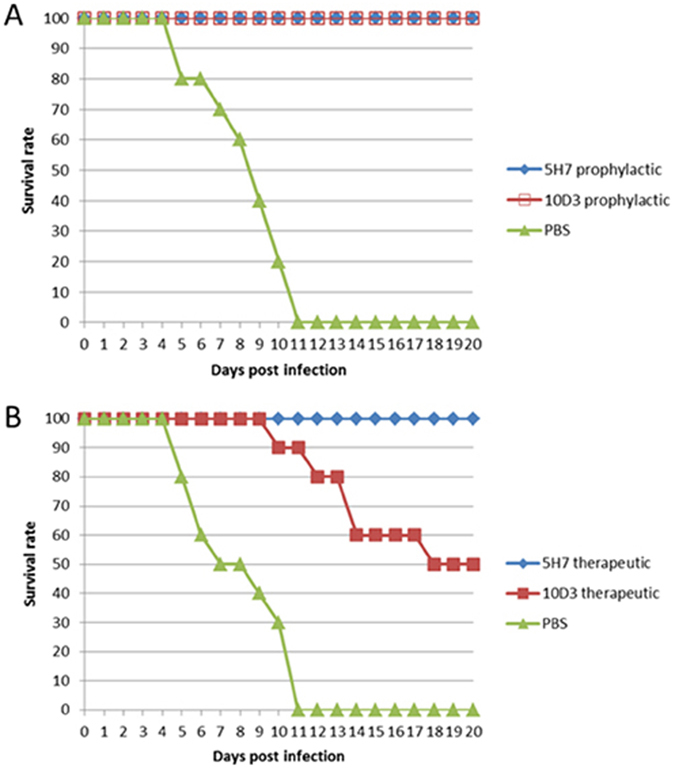
Prophylactic and therapeutic efficacy of Mab 5H7 and 10D3 against EV71 B4 infection of AG129 mice (**A**): Survival rate of mice in a prophylactic study. (**B**): Survival rate of mice in a therapeutic study. Survival and clinical signs of mice were monitored daily until death or until 20 dpi. PBS: represents infected group of mice without any treatment. n = 10.

To test the protective efficacy of 5H7 and 10D3, two-week old AG129 mice were subjected to both prophylactic (Fig. 8A) and therapeutic (Fig. 8B) studies with lethal challenge of EV71 WT B4 HFM41. In the control animals with virus infection only, 80% of mice developed severe limb paralysis as early as 6 dpi, and all of the mice died within 11 dpi. In contrast, the mice pre-treated with either 5H7 or 10D3 (administered at a dose of 10 μg per g of body weight) did not display any disease manifestations, and remained healthy throughout the experiment (Fig. 8A). However, in a therapeutic study in which antibodies were administered 24 h post-infection (Fig. 8B), only the 5H7-treated group showed 100% survival without any symptoms throughout the experiment. In the 10D3 therapeutic group, 70% of mice started to exhibit disease symptoms (e.g. limb paralysis) from 10 dpi, while 40–50% of mice died starting from 14 dpi.

**Figure 9:**
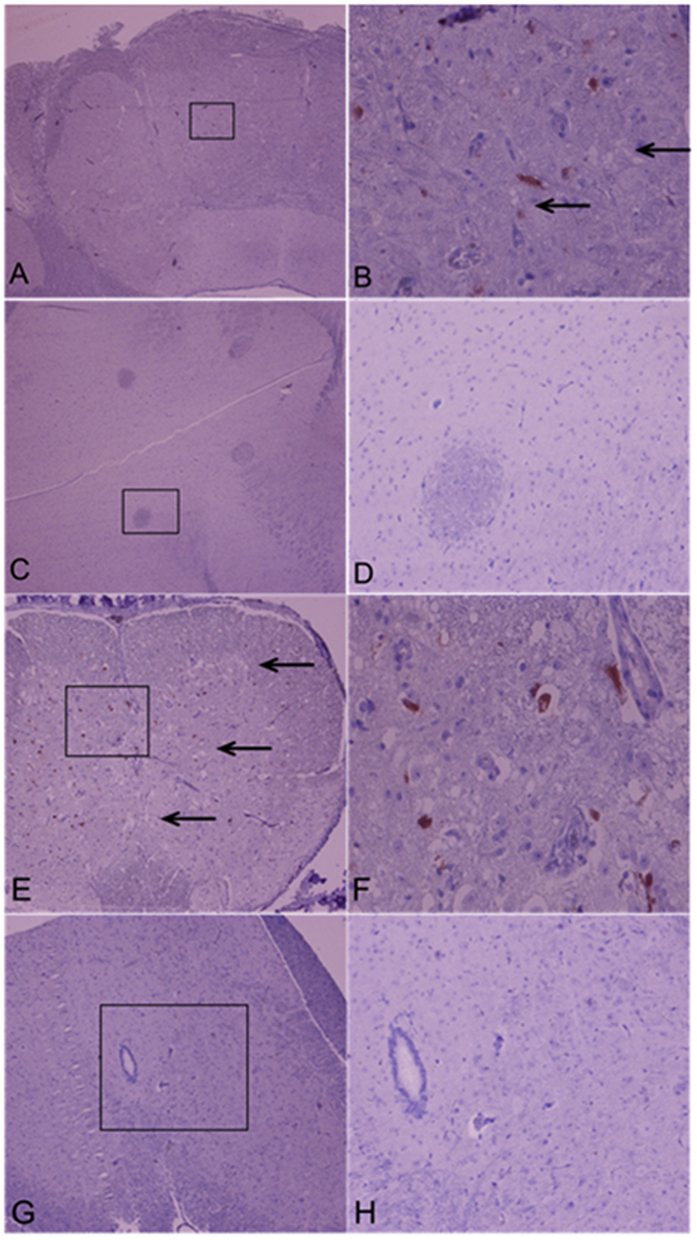
Immunohistochemistry and histopathology of EV71-infected AG129 mice without and with therapeutic protection with 5H7. Brain (**A–D**) and spinal cord (**E**,**F**) tissues were harvested and sectioned. Mice infected with a lethal dose of EV71, and treated with control antibody (**A,B,E,F**) displayed viral antigen and neuronal vacuolation. However, neither viral antigen nor neuronal lesion was observed in infected mice subjected to therapeutic protection with 5H7 (**C,D,G,H**). (**B,D,F,H**) Are views at higher magnification (200×) of (**A,C,E** and **G**) (40×).

To confirm the protective efficacy of 5H7, histopathologic examination of mouse brains and spinal cord was conducted. Mice from the control antibody group exhibited viral protein, neuronal vacuolation and neuronal loss in the brain and spinal cord. In contrast, no pathologic changes or viral antigen was observed in mice from the 5H7 therapeutic group. The intact brain and spinal cord tissue morphology (Fig. 9) suggest that Mab 5H7 confers effective passive protection against EV71 infection *in vivo*.

**Figure 10:**
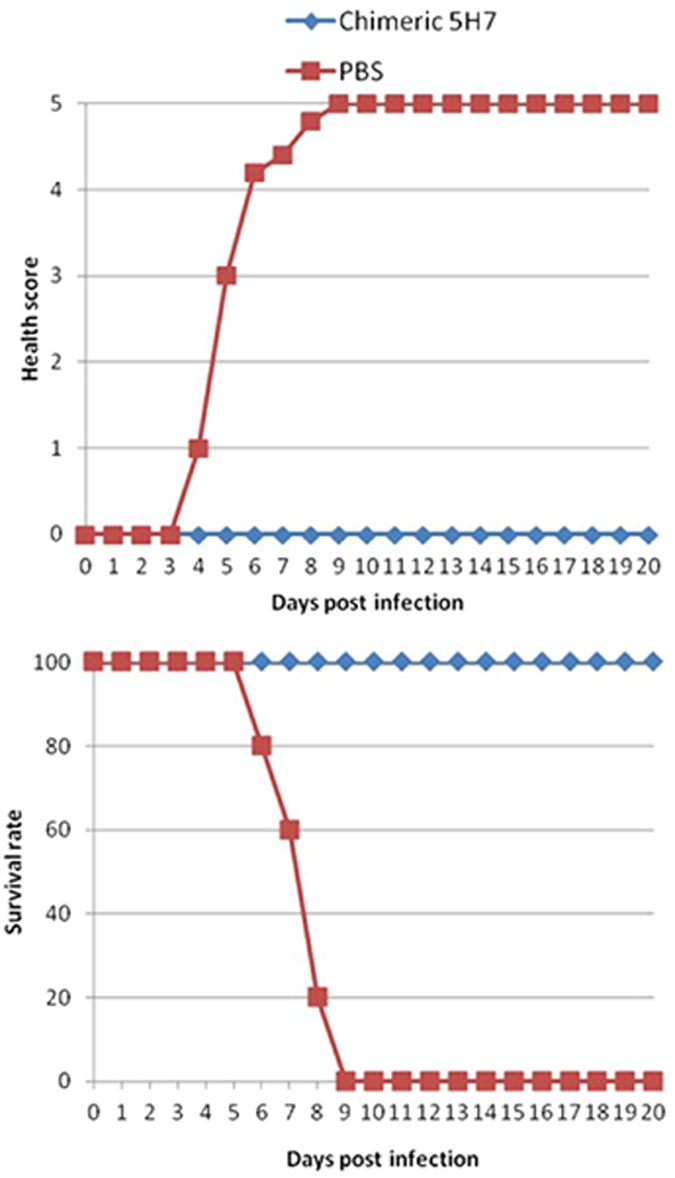
Therapeutic efficacy of chimeric 5H7 against EV71 B4 infection of AG129 mice. Clinical disease scores and survival rates were recorded and evaluated daily until death or until 20 dpi. PBS: represents infected group of mice without any treatment. n = 5.

Chimeric 5H7 with human and murine frame was constructed and expressed to explore the potential of this antibody for future human therapy. Therapeutic study was performed with chimeric 5H7 against B4 in the same mouse model (Fig. 10). Chimeric 5H7 at the same dose as murine 5H7 was able to protect 100% of mice from the lethal challenge with B4. No symptom was observed in the chimeric 5H7 group, while pathologic changes developed among untreated infected mice as recorded by health score. These findings indicate that chimeric 5H7 is able to confer therapeutic efficacy against EV71 *in vivo*.

## Discussion

Neutralizing monoclonal antibodies are specific antiviral agents that can be exploited for passive immunization of patients with acute infections. They offer advantages over conventional IVIG treatment with pooled human sera: (a) Therapies with Mabs reduce the risk of transmitting pathogens; (b) Mab products alleviate the problem of batch-to-batch variability; (c) The production of Mabs does not rely on the availability of donors. (d) Products with Mabs do not contain any non-neutralizing antibodies^26^. HFMD causes acute symptoms, and even severe outcomes including death in some patients^10^,^27^. Therefore, effective antibody therapy against HFMD requires specific monoclonal antibodies with broad-spectrum and high efficacy. In this study, Mab 5H7 was identified and characterized as a candidate that fulfills all the requisite advantages as an effective therapeutic antibody against EV71, including (a) cross-neutralizing activity against all genotypes; (b) efficient neutralization *in vivo*; (c) active efficacy in the form of human or chimeric antibody; (a) the IgG form for cost-effective large-scale production. The mechanism underpinning its neutralizing function was also revealed to better understand EV71 antibody therapy. In our previous study, 10D3 was identified as a novel neutralizing antibody against the VP3 “knob” region. In comparison, 5H7 not only possesses the same broad reactivity as 10D3, but also offers significant advantages over 10D3, making it an ideal EV71 therapeutics reagent.

5H7 is a broad neutralizing antibody targeting a conserved epitope in all EV71 genotypes. Similar to 10D3, 5H7 targets the VP3 protein of EV71. The mutation sites of the viral escape mutants of the two antibodies are adjacent to each other in the 3D structure. Besides these epitopes in the “knob” region, there are other neutralizing epitopes identified on VP3^28^. Unlike VP1, which is predominantly immunogenic^29^ but exhibits considerable variation among different genotypes, VP3 is well-conserved among different EV71 genotypes with amino acid identity of >97%, thus representing a good target of broadly neutralizing antibodies. According to BLAST analysis, the region spanning both epitopes of 10D3 and 5H7 (VP3 amino acids 59–75) is 100% conserved among all EV71 genotypes within GenBank. Furthermore, amino acids 72–74 (PVS) of the VP3 proteins of EV71 and bovine enterovirus are conserved across animal species^30^. In contrast to VP1^31^,^32^, antibody responses against VP3 are minor in humans upon EV71 infection. Therefore, the natural occurrence of mutants escaping from VP3 neutralizing antibodies is rare, and these mutations are unlikely to become dominant among EV71 strains under current antigenic selection. Besides, the generation of escape mutants *in vitro* requires the excess of antibody and long-term selection. In acute infections, over-dosage and long-term administration of therapeutic antibody in patients are not necessary for treatment against HFMD, which is not a chronic disease with prolonged symptoms. We believe that the conditions of viral population size and antibody concentration under which prophylactic antibody administration would typically occur are such that the probability of EV71 escape mutants occurring and coming to dominate the virus population would be relatively low^17^. Therefore, we suggest that Mabs 5H7, as well as 10D3, are worthy of further exploration as therapeutic or prophylactic agents for emerging strains of EV71. In addition, these antibodies might also be worthy of additional study for use in diagnostic applications.

5H7 blocks viral receptor binding for efficient neutralization, while 10D3 neutralization acts post-attachment. This may partly explain the difference in the performance of 5H7 and 10D3 as mouse therapeutic agents against EV71. 5H7 is able to neutralize EV71 before virus attachment by inhibiting virus binding to SCARB2 receptor. This activity of 5H7 provides both prophylactic and therapeutic efficacy in mice against EV71 lethal challenge. In contrast, 10D3 is also a VP3-targeting antibody but neutralizes virus post-attachment, which may account for the fact that 10D3 is functional prophylactically, but failed to confer sufficient protection in the therapeutic model. For antibody treatment against viral infectious diseases, antibodies that are capable of preventing virus infection are preferred. For example, for influenza treatment, neutralizing antibodies against hemagglutinin 1 (HA1)^33^,^34^ are widely used due to their efficient inhibition of virus attachment. In acute viral diseases, such as HFMD and H7N9 influenza^35^,^36^, symptoms develop rapidly. Although such antibodies are able to abrogate further virus replication and spread, they may not be efficient enough to thwart immediate disease effects following virus entry^37^. In comparison, antibodies blocking virus binding, such as 5H7, facilitate extracellular virus inactivation before the virus is able to cause any pathogenic effect on permissive host cells, culminating in successful therapeutic efficacy^38^. The difference in the therapeutic efficiency becomes evident especially when the antibodies are applied after virus challenge. Ideally, a cocktail of two types of antibodies is recommended for thorough virus clearance based on their different targets.

As an IgG antibody, 5H7 is suitable for efficient large-scale production and antibody humanization^33^. Due to the polymer structure, it is difficult to construct and express recombinant IgM antibody for humanization. In some cases, the activity of the expressed product is different from the original antibody and the preferred neutralizing function is lost after humanization^39^. An attempt was made to construct a chimeric IgG from 10D3 murine IgM. However, no neutralizing activity was detected against any EV71 virus with the chimeric 10D3 IgG, which impedes the clinic application of 10D3 and makes it impossible to compare the two antibodies in the same chimeric form at the moment. For any antibody intended for clinical use, humanization is eventually required to minimize unnecessary murine immunogenicity that may cause undesirable side-effects in patients^40^,^41^. In this study, a mouse-human chimeric 5H7 was constructed as a precursor for final humanization. The protective efficacy of this chimeric antibody was verified by virus neutralization (data not shown), and animal infection challenge of AG129 mice. Chimeric 5H7 possessed neutralizing activity as effective as the murine one both *in vivo* and *in vitro*. Using the 5H7 therapeutic strategy, 100% of mice survived the lethal virus challenge, indicating the successful conversion of 5H7. A completely humanized 5H7 will be expressed and characterized in a future study.

As an IgG antibody, 5H7 is more readily available than other isotypes in terms of ease in purification, long-term stability and greater neutralization efficiency^42^ thus making it an ideal therapeutic reagent for large-scale production to meet the urgent need posed by large HFMD outbreaks. Although several antibodies have been reported for application and animal trials against EV71^39^,^43^, the further development of these candidates are hindered either by failure in humanization or limited by unidentified mechanisms. Our findings have clarified the potential of 5H7 as an effective therapeutic antibody against EV71, i.e. relying on its conserved target epitope, IgG isotype and neutralization against virus attachment. 5H7 is effective for both prophylactic and therapeutic strategies in EV71-infected AG129 mice, while its chimeric 5H7 counterpart possesses similar efficacy against lethal challenge. In conclusion, this study not only characterizes a novel antibody for functional therapy against HFMD, but also reveals the mechanism underpinning its protective function, thus aiding future optimization and new technology for EV71 prevention and treatment.

## Additional Information

**How to cite this article**: Jia, Q. *et al*. Effective *in vivo* therapeutic IgG antibody against VP3 of enterovirus 71 with receptor-competing activity. *Sci. Rep.* **7**, 46402; doi: 10.1038/srep46402 (2017).

**Publisher's note:** Springer Nature remains neutral with regard to jurisdictional claims in published maps and institutional affiliations.

## Figures and Tables

**Table 1:** Neutralization titer with 5H7 (10 μg/ml) against representative strains of EV71 subgenogroups (100 TCID_50_).

Subgenogroup	Name	GenBank	Neutralization Titer Mab 5H7*
A	BrCr	U22521.1	32
B1	RG EV71-VP1 (B1)	EU703813.1 backbone	32
B2	7423/MS/87	U22522.1	32
B3	RG EV71-VP1(B3)	EU703813.1 backbone	32
B4	5865/SIN/000009	AF316321.2	32
B5	NUH0083/SIN/08	FJ461781.1	32
C1	Y90-3761	AB433864.1	64
C2	NUH0075/SIN/08	FJ172159.1	32
C3	RG EV71-VP1(C3)	EU703813.1 backbone	32
C4	75-Yamagata-03	AB177813.1	64
C5	3437/SIN/06	GU222654.1	32
A	EM S74L		<4
B4	EM S74L		<4
C2	EM S74L		<4
C4	EM S74L		<4
BR	RG S74L		<4

^*^Titers below 4 indicate negative neutralization activity.

**Table 2:** Alignment of VP3 proteins of different EV71 subgenogroups and escape mutants*.

R	N	L	L	E	L	C	Q	V	E	T	I	L	E	V	N	N	V	P	T	N	A	T	S	L	M	E	R	L	R	F	P	V	S	A	Q	A	G	K	G	Majority
								50								60								70								80								
R	N	L	L	E	L	C	Q	V	E	T	I	L	E	V	N	N	V	P	T	N	A	T	S	L	M	E	R	L	R	F	P	V	S	A	Q	A	G	K	G	A BrCr VP3.pro
R	N	L	L	E	L	C	Q	V	E	T	I	L	E	V	N	N	V	P	T	N	A	T	S	L	M	E	R	L	R	F	P	V	S	A	Q	A	G	K	G	B2 7423-MS-87 VP3.pro
R	N	L	L	E	L	C	Q	V	E	T	I	L	E	V	N	N	V	P	T	N	A	T	S	L	M	E	R	L	R	F	P	V	S	A	Q	A	G	K	G	B4 5865-SIN-000009 VP3.pro
R	N	L	L	E	L	C	Q	V	E	T	I	L	E	V	N	N	V	P	T	N	A	T	S	L	M	E	R	L	R	F	P	V	S	A	Q	A	G	K	G	B5_NUH0083_VP3.pro
R	N	L	L	E	L	C	Q	V	E	T	I	L	E	V	N	N	V	P	T	N	A	T	S	L	M	E	R	L	R	F	P	V	S	A	Q	A	G	K	G	C2 NUH007-SIN-08-VP3.pro
R	N	L	L	E	L	C	Q	V	E	T	I	L	E	V	N	N	V	P	T	N	A	T	S	L	M	E	R	L	R	F	P	V	S	A	Q	A	G	K	G	C4 yama lab strain VP3.pro
R	N	L	L	E	L	C	Q	V	E	T	I	L	E	V	N	N	V	P	T	N	A	T	S	L	M	E	R	L	R	F	P	V	L	A	Q	A	G	K	G	E1 5H7 + A VP3.pro
R	N	L	L	E	L	C	Q	V	E	T	I	L	E	V	N	N	V	P	T	N	A	T	S	L	M	E	R	L	R	F	P	V	L	A	Q	A	G	K	G	E2 5H7 + C2 VP3.pro
R	N	L	L	E	L	C	Q	V	E	T	I	L	E	V	N	N	V	P	T	N	A	T	S	L	M	E	R	L	R	F	P	V	L	A	Q	A	G	K	G	E3 5H7 + C4 VP3.pro
R	N	L	L	E	I	C	R	V	E	T	I	L	E	V	N	N	L	Q	S	N	E	T	T	P	M	Q	R	L	C	F	P	V	S	V	Q	S	K	T	G	CA16 U05876 VP3.pro

^*^The escape mutations are highlighted in the red box. While the EV71 strains displayed 100% homology in the VP3 region shown above, the amino acid sequence of CVA16 was distinctly different as shown in red letters.
